# l bnl compress: Lossy but not lossy compression, a python script to compress MX data

**DOI:** 10.1063/4.0000796

**Published:** 2025-10-27

**Authors:** Herbert J Bernstein, Jean Jakoncic

**Affiliations:** 1 Fresh Pond Research Institute, Cambridge, MA, USA; 2 Brookhaven National Laboratory, Upton, NY, USA

## Abstract

l_bnl_compress.py is a python script implementing the lossy compressions described in [1] for macromolecular crystallographic diffraction data. The lossy compressions use pixel-by-pixel binning, image-by-image summing, JPEG-2000 Daubechies (DB) wavelet compression from the movie industry, and HCompress Haar (now known as DB0) wavelet compression from astronomy which are combined with the usual lossless MX compressions [2].

The original version of the necessary software was based on the CBF [3] representation of the data and written in C and bash. However, much of the current pool of macromolecular crystallographic data, especially for Dectris Eiger detectors, is written in NeXus/HDF5 NXmx format [4]. The new version of the software, named l_bnl_compress.py is a python script using h5py [5] with the astropy module to provide access to HCompress [6] lossy compression and the glymur module provide access to JPEG-2000 lossy compression. The code is open source and accessible in GitHub.

We provide examples from three datasets: a lysozyme, a thermolysin, and an endonuclease immune effector. Future plans include a parallel version to facilitate real-time application, and integration with AI supervision to support module-by-module and resolution-sensitive choice of compression levels.
